# Notes and News

**Published:** 1894-08-25

**Authors:** 


### Notes and News.

Collected and Communicated.

The Linnean Society's gold medal, presented once in ten years, has been awarded to Dr. Ernst Haeckel for distinguished services in biology.

A medico-chirurgical society has been established at Pisa in connection with the University of that city. The transactions will be published every two months.

The distinguished professor of ophthalmology at Vienna, Professor Stellwag von Cariow, has resigned his post, at the age of 70. A meeting of his colleagues and pupils assembled to bid the professor good-bye from his professional career.

Out of nineteen candidates for the office, Dr. Stephen George Lloyd, of the Hospital Ships, Long Reach, Dartford, was elected Resident Medical Officer of the Swansea Hospital vice Dr. Cornelius A. Griffiths, resigned.

The annual meeting of the Sanitary Association was held this year at Ramsgate, Mr. Thomas, of Bermondsey, presiding. Mr. Tidman, a member of the Council, contributed a paper, and advocated the establishment of headquarters, a series of lectures, and a journal devoted to the interests of the association.

Prince and Princess Henry of Battenberg opened an extensive garden *fête* last week at Appuldurcombe Park, formerly the residence of the Earls of Yarborough, on behalf of the Royal National Consumption Hospital at Ventnor, and of its sister institution, the Royal Isle of Wight Infirmary and County Hospital at Ryde, which are both under the patronage of Her Majesty. Their Royal Highnesses were shown through the house, and afterwards went on to the lawn, where there were several tents in which special entertainments were given. In the evening the grounds were beautifully illuminated. The proceedings were repeated subsequently, and the result of the two days is expected to be a large addition to the funds of the hospitals on behalf of which the *fête* was devised.

The Corbett Hospital, at Brierley Hill, has been the scene of recent festivities, when the portrait of Mr. John Corbett, the founder of the hospital, was unveiled. The presentation is of special interest, as the movement entirely originated amongst the working population in recognition of the benefits Mr. Corbett has conferred upon them. The portrait, a life-sized one, by H. T. Wells, R.A., is now hung in the entrance hall of the hospital.

Edinburgh last week was the scene of the eighth International Ophthalmological Congress. About 150 members attended, amongst whom were many illustrious names of various nationalities. The president was Dr. Argyll Robertson. Dr. Knapp, of New York, Dr. Priestley Smith, Professor Leber, of Heidelberg, Professor Pflüger, of Berne, and Dr. Landolt, of Paris, all contributed papers. The Royal College of Physicians entertained the visitors at dinner, and many other entertainments were held in their honour.

The convalescent home presented by Mr. Passmore Edwards to the Working Men's Club and Institute Union of London, was opened at Pegwell Bay this month. The home has been fitted up from the old Clifton Hotel, and is arranged to accommodate twenty-five patients, with a possibility of extension. The building with alterations cost £3,500, and is provided with comfortable dining and recreation rooms, in addition to the sleeping accommodation. The community which will benefit by this generous gift numbers some 90,000 members, to whom the home will undoubtedly prove a great blessing.

The Board of Management of the Chelsea Hospital for Women has now elected a new medical staff in place of the gentlemen resigned. The Electing Board included Sir Spencer Wells and Mr. Jonathan Hutchinson and Dr. Robert Barnes. The numerical strength of the staff has been reduced, there being now two physicians for the in-patients in place of three, and four physicians to the out-patients instead of six. The gentlemen elected are: For the in-patients, Drs. W. Duncan, and W. H. Fenton; and to the out-patients, Drs. J. Inglis Parsons, R. O'Callaghan, A. E. Giles, and T. Watts Eden. With the exception of Drs. Duncan and O'Callaghan, the gentlemen appointed have already held office at the hospital.

A new Eye Infirmary has been opened this month at Greenock. It is the gift of Mr. Anderson Rodger, a shipbuilder, of Port Glasgow. There was a grand trades demonstration on the occasion, much interest being evinced in the event. The old Eye Infirmary was endowed with £6,000 by a citizen of Greenock, Mr. J. Furguson, fourteen years ago. The new building is built of terra-cotta, glazed brick, with red tiled roof. The waiting-room will accommodate one hundred persons. Adjoining it are surgeon's room, dispensary, and ophthalmoscope-room. There are two wards for male and female patients, with lavatories and bath-rooms attached. Dining and sitting rooms are adjacent. The upper floor is devoted to the officers' quarters, and here too are small private wards and a sitting-room. Comfort and efficiency have been provided for throughout, and the building has been secured at a cost of £2,000, and much of the furniture has been presented also.

